# *Helicobacter pylori* Infection Is Not Associated with Non-alcoholic Fatty Liver Disease: A Cross-Sectional Study in China

**DOI:** 10.3389/fmicb.2018.00073

**Published:** 2018-01-31

**Authors:** Nengguang Fan, Liang Peng, Zhenhua Xia, Lijuan Zhang, Yufan Wang, Yongde Peng

**Affiliations:** ^1^Department of Endocrinology and Metabolism, Shanghai General Hospital, School of Medicine, Shanghai Jiao Tong University, Shanghai, China; ^2^Department of Laboratory Medicine, Shanghai Songjiang Center Hospital, Shanghai, China; ^3^Department of Endocrinology, Shanghai Songjiang Center Hospital, Shanghai, China

**Keywords:** *Helicobacter pylori*, non-alcoholic fatty liver disease, lipid profile, gut microbiota, metabolic syndrome

## Abstract

**Background and Aim:** *Helicobacter pylori* infection has been reported to promote the development of a variety of extra-digestive manifestations, including type 2 diabetes, cardiovascular and liver diseases. Recently, the association between *H. pylori* infection and non-alcoholic fatty liver disease (NAFLD) was also proposed. However, evidence from different studies was controversial. We therefore performed this study to investigate the relationship between them in a large population of apparently healthy subjects in China.

**Methods:** A total of 21,456 subjects underwent a healthy checkup program were included. *H. pylori* infection was detected by 14C urea breath test (14C-UBT) and NAFLD was diagnosed by ultrasonography.

**Results:** Subjects infected with *H. pylori* had a more unfavorable metabolic profile, including higher levels of body mass index (BMI), blood pressure, triglycerides (TG) and lower levels of high-density lipoprotein cholesterol (HDL-C), as compared with those without *H. pylori* infection (all *P* < 0.05). Moreover, the prevalence rate of NAFLD was significantly increased in subjects with *H. pylori* infection when compared with those without *H. pylori* in women (23.6% vs. 21.5%, *P* < 0.05), but not in men (46.5% vs. 45.5%, *P* > 0.05). After adjusting for confounding factors including age, sex, BMI, blood pressure and lipid profiles, multivariate logistic analysis revealed that *H. pylori* infection was not independently associated with the risk of NAFLD in the total population (OR = 0.9, 95% CI = 0.9–1.0, *P* = 0.097). Also, subgroup analysis (stratified by age, sex, BMI, and diabetes status) showed no independent association between *H. pylori* infection and NAFLD.

**Conclusion:** Our data suggests that *H. pylori* infection is not independently associated with the risk of NAFLD in apparently healthy subjects.

## Introduction

Non-alcoholic fatty liver disease (NAFLD), defined as the presence of hepatic steatosis in the absence of alcohol use and other causes of liver disease, has become one of the most prevalent liver diseases worldwide (Hassan et al., 2014). It represents a spectrum of conditions from simple steatosis to non-alcoholic steatohepatitis (NASH) and cirrhosis. A growing body of evidence has linked NAFLD to obesity, dyslipidemia, diabetes and insulin resistance, and it has been considered to be a hepatic manifestation of metabolic syndrome (Fabbrini and Magkos, 2015).

*Helicobacter pylori* is a Gram-negative, spiral shaped pathogenic bacterium that colonizes the gastric epithelium in human population. The prevalence of *H. pylori* infection is about 30% in developed and up to 80% in developing countries. It has been demonstrated that *H. pylori* causes chronic gastritis, peptic ulcer disease, and gastric cancers (Wotherspoon et al., 1991; Wessler et al., 2017). Besides, persistent infection of *H. pylori* promotes immune cell infiltration and chronic inflammation, which induce the production and diffusion of pro-inflammatory cytokines and cause systemic effects. In recent years, *H. pylori* was reported to be associated with the development of a variety of extra-digestive manifestations, including type 2 diabetes, cardiovascular and liver diseases (Roubaud Baudron et al., 2013). Since *H. pylori* does not enter circulation, these extragastric manifestations are probably indirectly mediated by the inflammatory mediators produced by the infected gastric mucosa.

Recently, the relationship between *H. pylori* infection and NAFLD has also been investigated, while the results were controversial. Dogan et al. (2013) showed that fatty liver was significantly more frequent in *H. pylori*-positive patients. The severity of the fatty appearance assessed by ultrasonography was also higher in the *H. pylori*-positive group. Moreover, a cohort study conducted in Japan showed that subjects with *H. pylori* infection had a higher rate of incident NAFLD that those without *H. pylori* infection (Kim et al., 2017). In contrast, another study showed no association between *H. pylori* infection and NAFLD (Okushin et al., 2015). Moreover, *H. pylori* eradication was not found to affect liver fat content and liver function tests in NAFLD patients (Jamali et al., 2013). These studies had some limitations and further research is warranted to confirm the relationship between *H. pylori* infection and NAFLD. It also remains to be determined if *H. pylori* is implicated in the natural course of NAFLD, or if it is merely an incidental finding.

In the present study, we performed a cross-sectional investigation to determine whether *H. pylori* infection is associated with NAFLD in a large population of apparently healthy individuals in China. Moreover, the relationship between *H. pylori* and NAFLD was further investigated in subgroups stratified by age, sex, BMI, and diabetes status.

## Materials and Methods

### Subjects

The study population was recruited from adults who underwent health checkups at Songjiang branch of the Shanghai First People’s Hospital between May 2013 and June 2014. Trained physicians collected detailed information of demography, medical history and drinking status by standard questionnaires. Subjects with an alcohol intake >140 g/week for men and 70 g/week for women, a history of viral hepatitis, auto-immune hepatitis or other forms of chronic liver disease, a history of respiratory, heart failure or renal diseases were excluded from the study. Finally, a total of 28,171 subjects were included in analysis. Characteristics of the study subjects were summarized in **Table 1**. This study was approved by the Institutional Review Board of Shanghai First People’s Hospital affiliated to Shanghai Jiao Tong University School of Medicine. All participants were verbally informed about the study. Written informed consent was not required for this study because of observational nature of the study.

**Table 1:** Clinical and biochemical characteristics of the study subjects.

Variables	ALL	*H. pylori* negative	*H. pylori* positive	*P*
*N*	28,171	17,323	10,848


Sex (M/F)	14,389/13,782	8,537/8,786	5,852/4,996	<0.001


Age (years)	48.3 ± 15.0	48.2 ± 15.1	48.3 ± 14.9	0.581


BMI (kg/m^2^)	23.7 ± 3.2	23.5 ± 3.2	23.9 ± 3.3	<0.001


SBP (mmHg)	126.9 ± 19.6	126.4 ± 19.4	127.6 ± 20.0	<0.001


DBP (mmHg)	73.8 ± 11.2	73.4 ± 11.1	74.4 ± 11.5	<0.001


FPG (mM)	5.3 ± 1.0	5.4 ± 1.0	5.3 ± 1.0	0.788


HbA1c (%)	5.7 ± 1.1	5.7 ± 1.1	5.7 ± 1.0	0.304


TG (mM)	1.5 ± 1.2	1.4 ± 1.2	1.5 ± 1.2	0.032


TC (mM)	4.8 ± 0.9	4.8 ± 0.9	4.8 ± 0.9	0.052


LDL-C (mM)	2.9 ± 0.8	2.9 ± 0.8	3.0 ± 0.8	<0.001


HDL-C (mM)	1.5 ± 0.4	1.5 ± 0.4	1.4 ± 0.4	<0.001


UA (μM)	320.7 ± 86.8	318.8 ± 86.5	323.8 ± 87.2	<0.001


Scr (μM)	70.6 ± 20.9	70.1 ± 20.5	71.3 ± 21.5	<0.001


ALT (IU/L)	17 (12–24)	16 (12–23)	17 (12–24)	<0.001


AST (IU/L)	19 (16–23)	19 (16–22)	19 (16–23)	0.113




*Subjects were divided into two groups according to *H. pylori* infection status. Continuous variables were presented as means ± SD or median (interquartile range) Differences between two groups were tested by student’s ox*t*-test for continuous variables and *x*^*2*^ test for categorical variables. BMI, body mass index; SBP, systolic and diastolic blood pressure; DBP, diastolic blood pressure; TG, triglycerides, TC, total cholesterol, LDL-C, low-density lipoprotein cholesterol, HDL-C, high-density lipoprotein cholesterol; ALT, alanine aminotransferase; AST, aspartate aminotransferase; Scr, serum creatinine; UA, uric acid.*

### Anthropometric and Biochemical Measurements

All subjects were assessed after overnight fasting for at least 10 h. Body weight, height, systolic and diastolic blood pressure (SBP, DBP) were measured by an experienced physician. BMI was calculated as body weight in kilograms divided by body height squared in meters.

Blood samples were collected from the cubital vein by one experienced nurse. Fasting serum triglycerides (TG), total cholesterol (TC), low-density lipoprotein cholesterol (LDL-C), high-density lipoprotein cholesterol (HDL-C), alanine aminotransferase (ALT), aspartate aminotransferase (AST), serum creatinine (Scr), and uric acid (UA) were measured using an autoanalyzer (Beckman, Palo Alto, CA, United States). Blood glucose was measured with glucose oxidase method. HbA1c was determined by high-performance liquid chromatography (ARKRAY, Kyoto, Japan).

### *Helicobacter pylori* Infection Test

The diagnosis of *H. pylori* infection was based on the results of fasting 14C urea breath test (14C-UBT), which is one of the most important and reliable non-invasive approaches for detection of *H. pylori* infection. Subjects fasted for overnight were first given a tablet of urea labeled with an uncommon isotope of radioactive carbon-14. After 30 min, breath samples were collected and the amount of isotope labeled carbon dioxide was measured in exhaled breath by scintillation. A positive result indicated the existence of *H. pylori*.

### Diagnosis of NAFLD

The diagnosis of NAFLD was based on the results of abdominal ultrasonography using a high-resolution B-mode tomographic ultrasound system with a 3.5-MHz probe (Toshiba, Tokyo, Japan). According to Diagnostic Criteria of Nonalcoholic Fatty Liver Disease by the Chinese Society of Hepatology in 2010, hepatic steatosis was defined by the presence of at least two of three of the following abnormal findings: diffuse hyperechogenicity of the liver relative to the kidneys; attenuation of the ultrasound beam; poor visualization of intrahepatic architectural details. Alcohol consumption, viral, or autoimmune liver disease were determined by questionnaire survey and was excluded before NAFLD diagnosis.

### Statistical Analysis

All statistical analyses were performed using SPSS 13.0 (Chicago, IL, United States). Continuous variables were presented as means ± SD or median (interquartile range), and categorical variables were displayed as percentages (%). Non-normally distributed data were logarithmically transformed before analysis. Differences between two groups were tested by student’s *t*-test for continuous variables and *x*^2^ test for categorical variables. Logistic regression was also used to evaluate the association between *H. pylori* infection and NAFLD. *P* < 0.05 was considered statistically significant.

## Result

### Clinical Characteristics of the Study Population

Among the 28,171 enrolled individuals, 13,782 were women and 14,389 were men, and the mean (±SD) age was 48.3 ± 15.0 years. The overall prevalence rate of NAFLD was 34.3%. In comparison with women, men had a significantly higher prevalence of NAFLD (45.9% vs. 22.2%, *P* < 0.05).

In all subjects, 10,848 were infected with *H. pylori* (38.5%). Clinical and biochemical characteristics of the participants stratified by *H. pylori* infection status were summarized in **Table 1**. Subjects infected with *H. pylori* were more likely to be male, and had higher levels of body mass index (BMI) and blood pressure. In addition, more unfavorable lipid profiles were observed in the *H. pylori* infected group, including higher triglycerides (TG) and low-density lipoprotein cholesterol (LDL-C) levels and lower high-density lipoprotein cholesterol (HDL-C) levels as compared with those without *H. pylori* infection., Moreover, serum uric acid (UA), serum creatinine (Scr) and alanine aminotransferase (ALT), which represent the impairment of kidney or liver, were all elevated. In contrast, no significant difference was found in age, fasting blood glucose (FBG) and HbA1c between the two groups (**Table 1**), which suggests no impact of *H. pylori* infection on blood glucose.

### Prevalence of NAFLD According to the Infection of *H. pylori*

**FIGURE 1:**
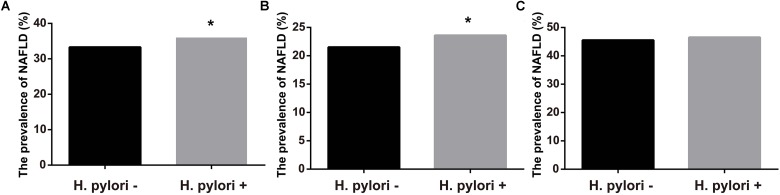
Prevalence of NAFLD according to infection of *H. pylori*. **(A)** Prevalence of NAFLD in all subjects. **(B)** Prevalence of NAFLD in female. **(C)** Prevalence of NAFLD in male. ^∗^*P* < 0.05.

**Figure 1** showed the prevalence of NAFLD according to *H. pylori* infection status. Those infected with *H. pylori* had a modest, but statistically significantly, higher prevalence of NAFLD when compared with the uninfected controls (36.0% vs. 33.3%, *P* < 0.05, **Figure 1A**). Further analyses stratified by sex showed that NAFLD prevalence in *H. pylori* infected participants were higher than those without *H. pylori* in women (23.6% vs. 21.5%, *P* < 0.05, **Figure 1B**). In contrast, no significant difference in the prevalence of NAFLD was observed between the two groups in men (46.5% vs. 45.5%, *P* > 0.05, **Figure 1C**).

### Association between *H. pylori* Infection and NAFLD

Logistic regression analysis was further performed to determine the independent association between *H. pylori* infection and risk of NAFLD. As shown in **Table 2**, *H. pylori* infection was associated with higher risk of NAFLD in univariate analysis (OR = 1.1, 95% CI = 1.1–1.2, *P* < 0.001, model 1). After adjustment for age and sex (model 2), the OR for NAFLD remained significant (OR = 1.1, 95% CI = 1.0–1.1, *P* < 0.004). However, when further adjusting for BMI, SBP and DBP (model 3), and plus FPG, HbA1c, TG, TC, HDL-C, LDL-C, and Scr (model 4), *H. pylori* infection was no longer associated with the risk of NAFLD (OR = 0.9, 95% CI = 0.9–1.0, *P* = 0.097) (**Table 2**).

**Table 2:** The risk of NAFLD according to the infection of *H. pylori.*

	*N*	OR	95% CI	*P*
Model 1	28,171	1.1	1.1–1.2	<0.001
Model 2	28,155	1.1	1.0–1.1	0.004
Model 3	26,521	0.9	0.9–1.0	0.097
Model 4	25,932	1.0	0.7–1.3	0.753


*Data are odds ratios (95% CI). Participants without *H. pylori* infection are defined as 0 and those with *H. pylori* infection as 1. N, number of subjects. Model 1 is unadjusted. Model 2 is adjusted for age, sex. Model 3 is further adjusted for BMI, SBP, and DBP. Model 4 is further adjusted for FPG, HbA1c, TG, TC, LDL-C, HDL-C, UA, and Scr.*

**FIGURE 2:**
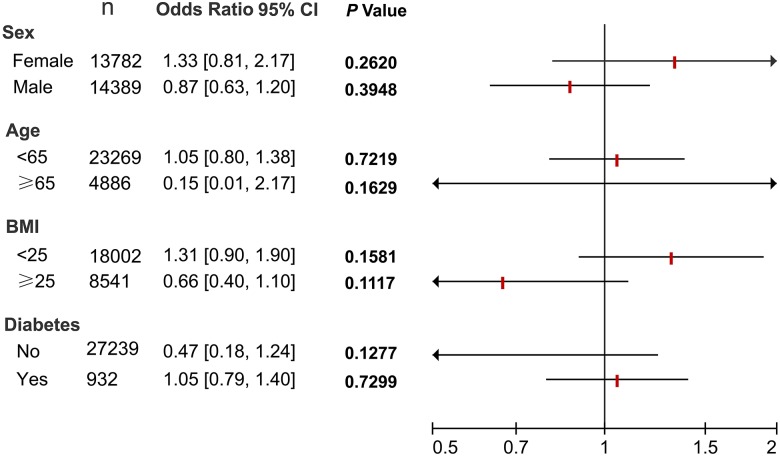
Subgroup analyses of the association between *H. pylori* and NAFLD. All 28,171 subjects were stratified by age, sex, BMI and diabetes status, respectively. Logistic regression analyses were then performed to determine the association between *H. pylori* and NAFLD after adjusting for confounding factors including age, sex, BMI, blood pressure, lipid profiles, and blood glucose. N, number of subjects.

Next, subgroup analyses were also performed. As shown in **Figure 2**, regardless of stratification by age, sex, BMI and diabetes status, no significant association between *H. pylori* and NAFLD was observed after adjusting for confounding factors.

## Discussion

*Helicobacter pylori* infection has shown to be correlated with NAFLD, but the results from different studies were controversial. In the present study of apparently healthy population, we found that *H. pylori* infection was related to metabolic risk factors including BMI, blood pressure, TG and HDL. However, no independent association between *H. pylori* and NAFLD was observed.

In the past few years, a growing body of evidence has shown an association between *H. pylori* infection and obesity and a more unfavorable metabolic profile (Xu et al., 2014; Baeg et al., 2016). Recently, in a cohort study including 17,028 adults, participants with *H. pylori* infection were found to have higher blood pressure, BMI, total cholesterol, LDL-C, triglycerides and HOMA-IR, and lower levels of HDL-C than subjects without *H. pylori* infection (Kim et al., 2017). Consistent to these previous studies, our present study showed significantly higher BMI, blood pressure, TG, LDL-C and UA levels in *H. pylori* group than the control group. Though the reasons responsible for the association between *H. pylori* and metabolic abnormalities have yet not been clearly elucidated, inflammation and insulin resistance induced by *H. pylori* has proposed to be the underlying mechanisms (Li, 2013; Vafaeimanesh et al., 2014). With regard to inflammation, peptidyl prolyl cis, *trans*-isomerase secreted by *H. pylori* was found to drive Th17 inflammation in gastric adenocarcinoma (Amedei et al., 2014). Interestingly, Th17 inflammation were found to play a pathogenic role in obesity and related inflammatory diseases. (Chehimi et al., 2017). So, whether Th17 inflammation mediates the association between *H. pylori* and metabolic abnormalities merits further investigation.

Non-alcoholic fatty liver disease has been recognized as a manifestation of metabolic syndrome. Recently, several previous studies have reported an association between *H. pylori* infection and NAFLD. Polyzos et al. (2013) found that *H. pylori* infection was more frequently observed in NAFLD patients than in healthy controls. Another study of 130 Japanese participants revealed that the prevalence of non-alcoholic steatohepatitis is higher in *H. pylori*-infected participants than in non-infected participants. More recently, a cohort study showed that *H. pylori* infection was significantly associated with the development of NAFLD, independent of metabolic and inflammatory risk factors (Kim et al., 2017). However, the link between *H. pylori* infection and NAFLD is still debated. A recent large-scale cross-sectional study including 13,737 Japanese adults reported that *H. pylori* infection is not associated with NAFLD (Okushin et al., 2015). Similarly, another cross-sectional study also found that *H. pylori* infection was not an independent risk factor of NAFLD (Baeg et al., 2016). More importantly, *H. pylori* eradication did not affect liver fat content, lipid profile, and insulin resistance in dyspeptic NAFLD patients (Jamali et al., 2013), arguing against a role of *H. pylori* in the development of NAFLD. In the present study, *H. pylori* infection was positively associated with NAFLD in univariate analysis. However, the association was no longer significant after controlling for BMI and blood pressure, suggesting the possibility that BMI mediate the association between *H. pylori* and NAFLD. In fact, as mentioned above, *H. pylori* infection is related to BMI and dyslipidemia, which are risk factors for NAFLD. The inconsistency of these studies may arise from different population enrolled and different diagnosis method of NAFLD. In addition, the methods of detecting *H. pylori* infection were different among studies. For example, in the cohort study performed by Kim et al. (2017), *H. pylori* infection status was assessed only with serum IgG to *H. pylori* measured by ELISA, while the serologic test cannot discriminate accurately between current and past infections. In contrast, in our and some other studies, urease breath test was performed to evaluate *H. pylori* infection (Baeg et al., 2016). Altogether, the association between *H. pylori* infection and NAFLD is remained to be determined in future studies, including clinical and basic researches.

Our study did not confirm the association between *H. pylori* and NAFLD. However, there are several limitations that require consideration. First, our study was cross-sectional, which did not allow to make a cause–effect inference. Second, the best method for an accurate diagnosis of NAFLD is liver biopsies. Ultrasonic examination, which was applied in the present study for diagnosis of NAFLD, is not sensitive enough to detect mild liver steatosis. Moreover, because hepatic ultrasound does not allow precise quantification of the severity of NAFLD, relationship between *H. pylori* infection and hepatic steatosis severity could not be evaluated. However, this non-invasive method is still widely used in clinical practice and epidemiological studies and is accepted for its sensitivity and specificity in detecting hepatic steatosis.

## Conclusion

The present study showed no independent association between *H. pylori* infection and NAFLD. More clinical and basic studies are required to further determine the relationship between them.

## Author Contributions

NF and YP conceived and designed the study. LP, ZX, and LZ collected the data. NF and YW analyzed the data. NF wrote the paper. All authors read and approved the manuscript.

## Conflict of Interest Statement

The authors declare that the research was conducted in the absence of any commercial or financial relationships that could be construed as a potential conflict of interest.
